# Evaluation the -174G>C Genetic Polymorphism of Interleukin-6 in Iranian Patients with Chronic Lymphocytic Leukemia

**DOI:** 10.30699/IJP.2023.544600.2790

**Published:** 2023-10-15

**Authors:** Zakieh Rostamzadeh Khameneh, Mahshid Mohammadian, Ali Eishi Oskuie, Rahim Asghari, Mohadeseh Nemati

**Affiliations:** 1 *Solid Tumor Research Center, Urmia University of Medical Sciences, Urmia, Iran*; 2 *Department of Clinical Biochemistry, School of Medicine, Urmia University of Medical Sciences, Urmia, Iran*; 3 *Department of Hematology and Medical Oncology, Urmia University of Medical Sciences, Urmia, Iran*; 4 *Department of Internal Medicine, School of Medicine, Solid Tumor Research Center, Urmia University of Medical Sciences, Urmia, Iran*

**Keywords:** Chronic lymphocytic leukemia, Interleukin-6, Polymorphism

## Abstract

**Background & Objective:**

Interleukin-6 (IL-6) is involved in inflammation and has a significant role in chronic lymphocytic leukemia (CLL) progression. Accordingly, IL-6 level may increase in CLL-affected patients compared to healthy individuals. The -174G>C single nucleotide polymorphism (SNP) in IL-6 promoter region has been related to differences in IL-6 transcription. Therefore, we investigated the possible association of IL-6 polymorphism with CLL.

**Methods:**

We examined the -174G>C SNP in *IL-6* gene and studied its possible relationship with CLL in affected patients and in healthy controls using Amplification Refractory Mutation System- polymerase chain reaction genotyping method. IL-6 plasma level was measured in both studied groups.

**Results:**

According to the results, IL-6 mean plasma concentration was increased significantly in the CLL patients compared to the controls. However, 174G>C genotype of the *IL-6* gene was not associated with CLL. Furthermore, there were no significant differences in the distribution of allele and genotype frequencies between the CLL-affected patients and the controls (*P*>0.05).

**Conclusion:**

Our study showed that -174G>C SNP in promotor of *IL-6* gene could not be considered a risk factor for CLL. Larger prospective studies should be performed to confirm our results.

## Introduction

The B-cell chronic lymphocytic leukemia (B-CLL) cells growth is associated with exogenous growth factors (in vitro) (1). Cytokines as small proteins are produced by various cells. Indeed, cytokines have a diverse range of physiological functions, for example, in immune system. In this regard, functional pleiotropy is one of the features of these proteins. Cytokines have a regulatory function in proliferative activities and cell differentiation based on the cell type. Various types of cytokines encompass interferons, growth factors, and interleukins (2). 

 Interleukin-6 (IL-6) as a multifactorial cytokine may have a role in the severity and progression of numerous types of cancers. Various observational studies have proposed that circulating IL-6 can describe inter-individual variability in cancer predisposition (3). 

Considering that plasma levels of IL-1 beta, IL-6, and IL-1Ra have been proposed to partially rely on gene polymorphism, the formerly described polymorphisms of these genes and the *IL-6 *gene were investigated (1).

Some common polymorphisms of cytokine genes are involved in the Th1/Th2 balance and inflammatory response and may have a role in the CLL progression (4).

 In this regard, IL-6 (a 23.7-kDa pleiotropic cytokine) is produced by immune cells, adipose tissue, and cardiovascular components. This cytokine primarily functions in the progression of inflammation, as well as functioning as an endogenous pyrogen in patients with infection (5, 6). Several studies have focused on the IL-6 promoter region since various polymorphisms including −598A/G, −572C>G, −597G/A, and −174G>C are recognized in this region. Among these polymorphisms, −572C>G and −174G>C are the most investigated ones (5).

 Considering the above-mentioned, we aimed to evaluate the −174G>C single-nucleotide polymorphism (SNP) in *IL-6* gene, its possible susceptibility to CLL, and also its probable effect on IL-6 plasma levels. To this end, we comparatively evaluated the -174G/C SNP of IL-6 in Iranian CLL-affected patients and the controls. 

## Material and Methods

**Demographic Data and Blood Sampling**

In order to evaluate the -174G/C SNP in the promoter region of *IL-6* gene, 35 CLL patients and 34 healthy controls from the hospitals affiliated to Urmia University of Medical Sciences, Iran, were included in this case-control study. The study protocol was reviewed and approved by Urmia University of Medical Sciences (Code of Ethics: IR.UMSU.REC. 1395.272).

Patients were aged 27-64 years with a history of confirmed CLL who had referred to the educational hospitals of Urmia University of Medical Sciences, between January and September 2019 and were recruited as the patient group. CLL patients with other disorders were excluded from this study. Demographic information (age, gender, etc.) of the subjects in both study and control groups were collected.

Based on an oncologist diagnosis, 35 CLL patients and 34 healthy controls were considered for further analysis. Then in the CLL patients, CD19, CD5, and CD23 markers were evaluated using immunophenotyping (7) (Table 1). Blood samples were collected in anticoagulant-containing tubes, centrifuged, and their plasma were isolated. The plasma levels of IL-6 were measured for both groups using the enzyme-linked immunosorbent assay (ELISA).

**Table 1:** Immunophenotyping of various CD markers in the CLL patients

Immunophenotyping	Percent
CD2	4.5%
CD3	9.1%
CD5	77.3%
CD10	9.1%
CD19	54.5%
CD20	86.4%
CD23	77.3%
CD25	9.1%
CD38	54.5%
CD79	86.4%
CD116	4.5%
BCL2	4.5%

**Genomic DNA Extraction and ARMS-PCR for −174 G>C SNP Detection**

**Fig. 1:**
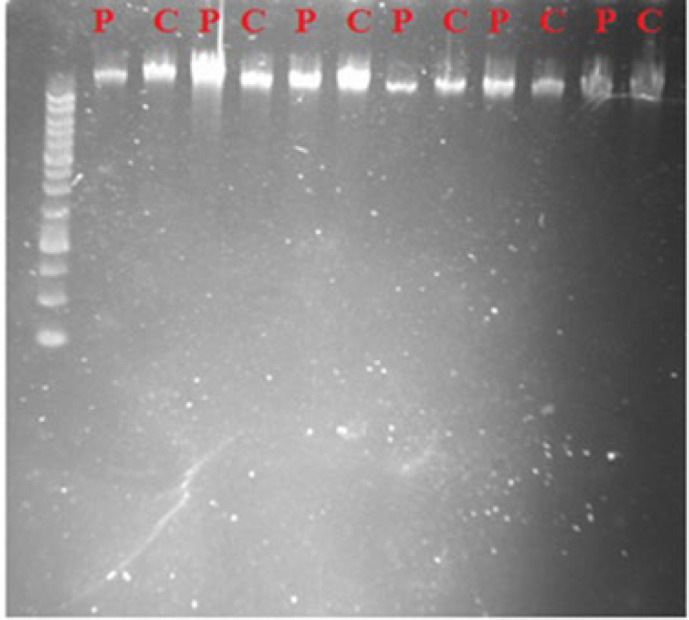
Extracted DNA from the CLL patients and healthy control individuals visualized on 1% agarose gel electrophoresis. P; patient. C; Control

The blood samples (2 mL) obtained from the patients and controls were subjected for the genomic DNA extraction using the manufacturer’s protocol [DNA Purification Kit, GeneAll]. DNA was isolated from the peripheral blood leukocytes to identify the −174G>C gene polymorphism. The extracted DNA was visualized on agarose gel 1% (Figure 1). To detect the SNP, Amplification Refractory Mutation System- polymerase chain reaction (*ARMS*-*PCR*) genotyping method was employed (using specific pairs of primers; Table 2).

 ARMS-PCR reaction was carried out in a total 20-μL volume comprising DNA sample, forward and reverse primers, dNTP (0.3 μL), MgCl2, Pfu enzyme, and ddH_2_O. The PCR amplification was performed as follows: initial denaturation at 94°C for 10 min, 40 cycles of denaturation at 94°C for 45 s, annealing at 58°C for 45 s, and extension at 72°C for 45 s, and final extension at 72°C for 7 min. 

The products of ARMS-PCR were visualized on Agarose gel 1%, and were compared to the 100-bp DNA Ladder (Thermo Scientific). Then, the odds ratios were utilized for investigating the relationships between IL-6 genetic polymorphism and CLL (with corresponding 95% confidence intervals).

**Statistical Analysis**

Statistical analyses were performed using the IBM SPSS software 22 (SPSS Inc., Chicago, Ill., USA) and applying the Pearson Chi-Square test. Indeed, Chi-square test was utilized to compare the genotype and allele frequencies of the −174G>C SNP between the cases and controls. A P-value <0.05 was considered as the significance level. Homozygosis and heterozygosis were directly counted in the control and patient groups

## Results

 To assess the 174G>C polymorphism in the promotor region of *IL-6* gene, genomic DNA was extracted from blood samples of the patients and controls and evaluated by ARMS-PCR method. The quality of extracted DNA was confirmed by Agarose gel 1% (Figure 1). The immunophenotyping of various CD markers in the CLL patients are presented in Table 1. Based on the results (Table 3), in subgroup division of patients versus controls, no significant differences were observed in the genotype and allelic distributions between the CLL and control groups (*P*>0.05). 

**Fig. 2:**
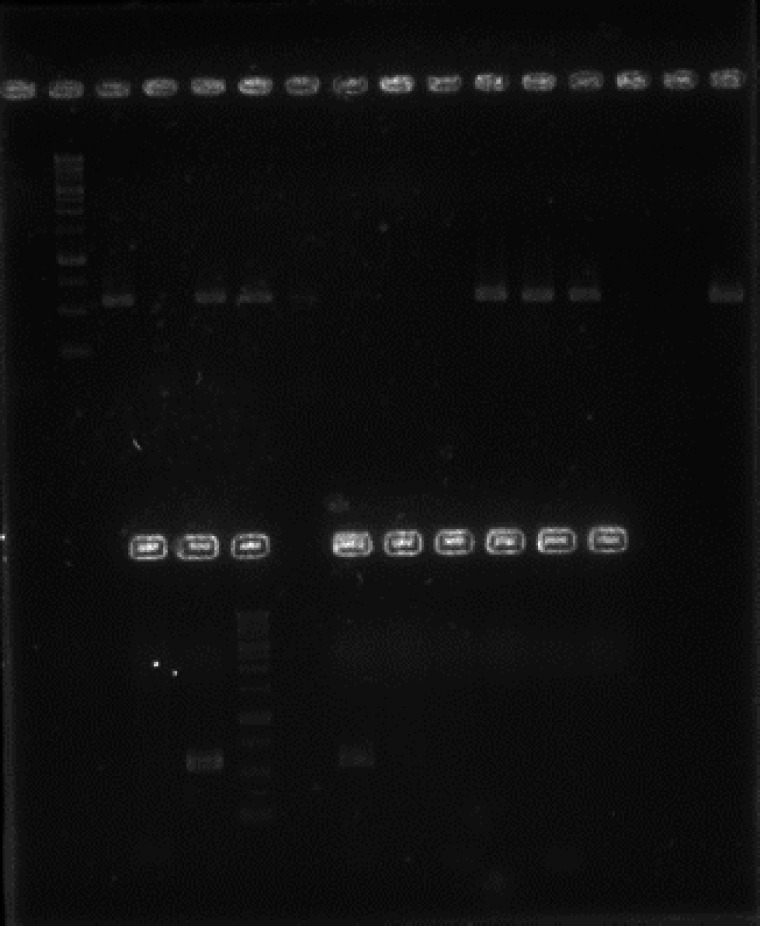
The PCR product Band pattern of IL6 -174F2 and IL6-174R (upper gel) and IL6-174F1 and IL6-174R. M: 1kb marker**.** From the left side of gel, the odd numbers from one indicate the patients and the even numbers indicate the controls

After performing ARMS-PCR, the PCR products were visualized on Agarose gel 1% (Figure 2).

**Table 2:** Specific primers for evaluation of the −174 G>C *SNP in *promoter of IL-6 gene by ARMS-PCR method

Number	Primer Name	Sequence	Product size (bp)
1	IL6-174F1	CACTTTTCCCCCTAGTTGTGTCTTGCC	527
	IL6-174R	ATTCGTTCTGAAGAGGTGAGTGGC	
2	IL6-174F2	CACTTTTCCCCCTAGTTGTGTCTTGCG	527
	IL6-174R	ATTCGTTCTGAAGAGGTGAGTGGC	

Based on our findings, there was no significant differences (*P*>0.05) in the frequency of IL-6, -174 GG genotype and G allele (*P*>0.05) between the CLL group and the controls. Furthermore, there was no significant increase in the distribution of IL-6 GG genotypes, and an insignificant reduction was observed in the frequency of GC genotype (*P* >0.05) between the CLL patients and the control subjects (Table 3). Moreover, there was no significant difference in the IL-6 (-174C/G) SNP frequencies between the CLL and control individuals.

According to our results, the mean IL-6 plasma level was 1.4 pg/mL in the control group. While, the CLL patients had a mean plasma IL-6 level of 7.35 pg/mL.

The mean serum concentration of IL-6 was increased significantly in the CLL cases compared to the controls (*P*<0.05).

In our study, the increased IL-6 level was not associated with some studied parameters including the patient age and white cell count. 

**Table 3:** Genotype and allelic distributions of -174G>C Polymorphism in the CLL patients and controls by ARMS-PCR method

Genotype distribution	Patient N=33	Control N=34	P-value	Patient (male) N=18	Control(male) N=14	P-value	Patient (female) N=15	Control(female) N=20	P-value
**GG**	24(72.72%)	22(64.7)	0.308	13(72.22)	8(57.14)	0.311	11(73.33%)	14(70%)	0.225
**GC**	5(15.15%)	10(29.41)		2(11.11%)	4(28.57)		3(20%)	6(30%)	
**CC**	4(12.12%)	2(5.88%)		3(16.66)	2(14.28%)		2(13.33)	-	
Allelic distribution	**Patient N=66**	**Control** **N=68**	**P-value**	**Patient (male)** **N=36**	**Control(male)** **N=28**	**P-value**	**Patient (female)** **N=30**	**Control(female)** **N=40**	**P-value**
**G**	53(80.3)	54(79.41%)	0.379	28(77.77)	20(71.42%)	0.361	25(83.33%)	34(85%)	0.344
**C**	13(19.69)	14(20.58)		8(22.2)	8(28.57)		5(16.6)	3(15%)	

## Discussion

CLL is considered as one of the prevalent types of leukemia in adults and is recognized by the increase in mature type B lymphocytes in lymphatic organs and peripheral blood (7).

IL-6 is the main risk factor involved in tumorigenesis. Nevertheless, earlier studies on the relationships of polymorphisms in IL-6 promoter through predisposition to various cancers are rather contrary (8). Indeed, the *IL-6* gene is recognized as a pro-inflammatory cytokine and has a significant function in the pathogenesis of numerous cancer types (8).

Stimulation of STAT3 and NF-κB is supported by IL-6. Once stimulated, two transcription elements regulate the gene expression of factors involved in the cancer cells’ growth (9).

*IL-6* gene promoter comprises several regulatory sites associated with the gene expression induction. In the promoter and encoding sequence, various SNPs are found. One of these SNPs is -174G/C, at position -174 of the promoter region [C (cytosine) to G (guanine) transition] (10). 

It has been indicated that the -174G/C SNP of the IL-6 promoter affects the transcription rate of IL-6 (11, 12).

The -174G/C polymorphism may be related to the inter-individual variations in the transcription and expression of the *IL-6* gene, thereby influencing the vulnerability to various disorders (13).

It has been proposed that IL-6 SNP is related to the prognosis of some cancers such as breast cancer, bladder cancer, neuroblastoma, and non-small cell lung cancer (14-19). Although the relationship of -174G>C SNP in the *IL-6* gene to various cancers has been evaluated, these results are contradictory (19).

Consequently, we conducted this case-control study to evaluate the relationship between the SNP in promoter region of *IL-6* gene and CLL. Our results displayed that there was no significant difference in the frequencies of IL-6 promoter SNP (-174G>C) between the healthy controls and CLL patients. Additionally, the subgroup analysis showed that -174G>C polymorphism distribution was not significantly different between the two studied groups. 

In this study, the plasma levels of IL-6 were increased in the CLL group compared to the controls. Likewise, in a study by Hulkkonen *et al.*, IL-6 plasma concentrations in the B-CLL patients were increased compared to the healthy subjects, corroborating our results. The authors also evaluated the polymorphism in the *IL-6* gene. They indicated that the allele frequencies of the studied genes were comparable in the controls and patients. They indicated that some mechanisms except for allelic imbalance of their genes were the main reasons for the profiles of cytokine detected in this disease (1). 

Definitely, increased levels of IL-6 were associated with overall survival and prognostic factors of CLL (20).

 Recent studies have shown that in the CLL patients, plasma levels of IL-6 were elevated. The level of IL-6 is increased in a disease stage-dependent manner (1, 20, 21).

It has been reported that serum IL-6 levels were increased in the CLL patients compared to the control individuals (22). 

 Based on another report**, **IL-6 produces both growth-enhancing and differentiation-prompting signals. In this regard, in M1 cells, Stat3 might be a main molecule that regulates the cellular decision from cell growth to differentiation in in M1 cells (23).

In another study by Belluco *et al.*, the effects of -174G>C polymorphism on serum level of IL-6 were found in colorectal cancer patients (24). In other study by Mandal *et al.*, a relationship was detected between the -174G/C polymorphism and prostate cancer (25). These results (24, 25) were not in line with our data. These disagreements might be explained by the differences in studied populations. 

In another study by Liu *et al.* (26) in 2020, IL-6 (rs2069830) and polymorphisms of *IL-10* gene were evaluated in specimens obtained from acute lymphoblastic leukemia-affected children and healthy children. These gene polymorphisms were related to the pathogenesis of childhood acute lymphoblastic leukemia. This result (26) was not similar to our results in that this difference may be related to the demographic parameters of studied populations and type of leukemia. 

Likewise in a similar study in 2010, the serum levels of IL-6 were evaluated in 30 myeloid leukemia patients versus controls. The results showed that in the myeloid leukemia patients, serum levels of IL-6 were different from those of the controls (27). 

Furthermore, in a similar study in 2000, on meyloma patients (73 patients), 27 subjects with gammopathy of undetermined significance and 129 healthy controls were included. The results showed that the *IL-6* gene polymorphisms did not associate with multiple myeloma susceptibility (28). 

We did not find any exact similar study to compare with those found in our study. According to our results, the mean plasma IL-6 level was significantly increased in the CLL patients compared to the healthy controls. Moreover, the −174G>C genotype of *IL-6* gene was not related to CLL. There was no difference in the distribution of −174G>C SNP in the CLL cases compared with the controls (*P*>0.05). Distribution of the GG genotype was not different between the CLL and healthy groups (*P*>0.05). In addition, the IL-6 GC genotype was not linked to the CLL. 

## Conclusion

We evaluated frequencies of the −174G>C SNP and its possible effect on the levels of IL-6 as a pro-inflammatory cytokine. Our results revealed that there was no association between the −174G>C polymorphism in the *IL-6* gene and CLL. These findings suggested that predisposition of genetic differences in the *IL-6* gene might not be associated with the increased risk of CLL in this studied population. The IL-6 polymorphisms may not have a role in CLL susceptibility in the studied patients. Overall, our preliminary study displayed that G allele and GG genotype of IL-6 (-174G/C) could not be considered as CLL-related risk factors. Larger prospective studies should be conducted to confirm these results.

## Funding

This study supported financially by Urmia University of Medical Sciences.

## Conflict of Interest

None.
